# ABCB1 and ABCG2 Together Limit the Distribution of ABCB1/ABCG2 Substrates to the Human Retina and the *ABCG2* Single Nucleotide Polymorphism Q141K (c.421C> A) May Lead to Increased Drug Exposure

**DOI:** 10.3389/fphar.2021.698966

**Published:** 2021-06-16

**Authors:** Myriam El Biali, Rudolf Karch, Cécile Philippe, Helmuth Haslacher, Nicolas Tournier, Marcus Hacker, Markus Zeitlinger, Doreen Schmidl, Oliver Langer, Martin Bauer

**Affiliations:** ^1^ Department of Clinical Pharmacology, Medical University of Vienna, Vienna, VIE, Austria; ^2^ Centre for Medical Statistics, Informatics, and Intelligent Systems, Medical University of Vienna, Vienna, VIE, Austria; ^3^ Division of Nuclear Medicine, Department of Biomedical Imaging and Image-guided Therapy, Medical University of Vienna, Vienna, VIE, Austria; ^4^ Department of Laboratory Medicine, Medical University of Vienna, Vienna, VIE, Austria; ^5^ Laboratoire d’Imagerie Biomédicale Multimodale (BioMaps), CEA, CNRS, Inserm, Service Hospitalier Frédéric Joliot, Université Paris-Saclay, Orsay, France

**Keywords:** ABCG2, ABCB1, blood-retinal barrier, c421C>A, single-nucleotide polymorphism, PET, human, tariquidar

## Abstract

The widely expressed and poly-specific ABC transporters breast cancer resistance protein (ABCG2) and P-glycoprotein (ABCB1) are co-localized at the blood-brain barrier (BBB) and have shown to limit the brain distribution of several clinically used ABCB1/ABCG2 substrate drugs. It is currently not known to which extent these transporters, which are also expressed at the blood-retinal barrier (BRB), may limit drug distribution to the human eye and whether the *ABCG2* reduced-function single-nucleotide polymorphism (SNP) Q141K (c.421C > A) has an impact on retinal drug distribution. Ten healthy male volunteers (five subjects with the c.421CC and c.421CA genotype, respectively) underwent two consecutive positron emission tomography (PET) scans after intravenous injection of the model ABCB1/ABCG2 substrate [^11^C]tariquidar. The second PET scan was performed with concurrent intravenous infusion of unlabelled tariquidar to inhibit ABCB1 in order to specifically reveal ABCG2 function.In response to ABCB1 inhibition with unlabelled tariquidar, *ABCG2* c.421C > A genotype carriers showed significant increases (as compared to the baseline scan) in retinal radiotracer influx *K*_1_ (+62 ± 57%, *p* = 0.043) and volume of distribution *V*_T_ (+86 ± 131%, *p* = 0.043), but no significant changes were observed in subjects with the c.421C > C genotype. Our results provide the first evidence that ABCB1 and ABCG2 may together limit the distribution of systemically administered ABCB1/ABCG2 substrate drugs to the human retina. Functional redundancy between ABCB1 and ABCG2 appears to be compromised in carriers of the c.421C > A SNP who may therefore be more susceptible to transporter-mediated drug-drug interactions at the BRB than non-carriers.

## Introduction

The transport of specific molecules across lipid membranes is an essential function of all living organisms (Liu, 2019). In humans, the widespread expression and poly-specificity of the adenosine triphosphate-binding cassette (ABC) family efflux transporter breast cancer resistance protein (ABCG2) makes it an important determinant of the pharmacokinetics of a variety of drugs (Mao and Unadkat, 2015). Many ABCG2 substrates are additionally substrates of another ABC-transporter, P-glycoprotein (ABCB1), so that the net effect on the disposition of drugs which are dual ABCB1/ABCG2 substrates may be attributed to the combined action of both transporters. These two transporters have been recognized by the International Transporter Consortium to be involved in clinically relevant transporter-mediated drug-drug interactions (DDIs) given their impact on the disposition of their substrates (International Transporter et al., 2010). The co-localization of ABCG2 and ABCB1 at several blood-tissue barriers suggests a crucial role in protecting key vulnerable and/or target tissues, e.g., the brain or the placenta, from xenobiotics and harmful metabolites. It is, however, difficult to predict the functional impact of ABCB1- and ABCG2-mediated efflux on tissue exposure from conventional plasma pharmacokinetic data (Wijaya et al., 2017).

ABCB1 and ABCG2 are co-expressed at the luminal membrane of brain capillary endothelial cells contributing to the protective function of the blood-brain barrier (BBB) (Uchida et al., 2011; Kalvass et al., 2013; Billington et al., 2019; Li and Zhu, 2020). Studies in *Abcb1a/b* and *Abcg2* knockout mice have provided evidence for functional redundancy between ABCB1 and ABCG2 in limiting the distribution of dual ABCB1/ABCG2 substrate drugs to the brain. In absence of either ABCB1 alone or ABCG2 alone (*Abcb1a/b* ^*(−/−)*^ mice or *Abcg2* ^*(−/−)*^ mice) the remaining transport capacity of the other transporter was largely sufficient to restrict brain distribution of ABCB1/ABCG2 substrates, for which brain distribution was only substantially increased in absence of both transporters (*Abcb1a/b* ^*(−/−)*^ *Abcg2* ^*(−/−)*^ mice) (Kodaira et al., 2010; Wijaya et al., 2017; Robey et al., 2018). A comparable functional redundancy between ABCB1 and ABCG2 has been confirmed *in vivo* at the human BBB (Bauer et al., 2016). Transporter-mediated DDIs at the BBB may potentially result in cerebral uptake and toxicity of medications that normally are not targeted to the brain without significant changes in drug plasma concentrations (Sasongko et al., 2005; Eyal et al., 2009; Bauer et al., 2017), although the risk for their occurrence in clinical practice is considered relatively low (Kalvass et al., 2013).

The eye, just like the brain, is a vulnerable organ as it requires the strict maintenance of a stable inner environment to insure neuro-retinal homeostasis and its sensory function (Fujii et al., 2014). The protection of the posterior segment of the eye, especially the retina, from systemically circulating phototoxic endogenous and exogenous substances and the regulation of the influx transport of vital molecules are essentially provided by the blood-retinal barrier (BRB) (Asashima et al., 2006; Agrahari et al., 2016), in a similar way as the BBB does for the brain (Fujii et al., 2014). The BRB is divided in two layers: (a) the inner BRB consisting of endothelial cells of the retinal capillaries (ECRC) and (b) the outer BRB composed of the retinal pigmental epithelial (RPE) cells, located between the neural retina and choriocapillaris.

There is evidence from preclinical studies that both, ABCB1 and ABCG2, are expressed at the BRB. ABCG2 was identified in mouse and rat retina and in the conditionally immortalized rat ECRC cell line TR-iBRB (Asashima et al., 2006) as well as concomitantly with ABCB1 at the luminal side of ECRC in rabbit and mouse eyes (Chapy et al., 2016; Pascual-Pasto et al., 2017). The data concerning the expression of ABCG2 and ABCB1 at the outer BRB are contradictory (Liu and Liu, 2019). A dominant protein expression of ABCG2 in pig eyes at the inner BRB over the outer BRB (22.8 fmol/μg protein and 2.76 fmol/μg protein respectively) was demonstrated (Zhang et al., 2017). The same study revealed that ABCG2 expression at the inner porcine BRB is 2.6-fold higher than that of ABCB1 and that the transporter expression pattern is positively correlated between the BBB and the inner BRB in pigs (Zhang et al., 2017). These findings are consistent with absolute quantification data at the human BBB, which showed a higher expression of ABCG2 than of ABCB1 (Uchida et al., 2011; Billington et al., 2019; Li and Zhu, 2020).

For the human BRB, ABCG2 and ABCB1 protein expression has been corroborated with a predominance of ABCG2 through RNA expression profiling and immunohistochemistry (Dahlin et al., 2013). All in all, it is currently not known to which extent these two efflux transporters limit the distribution of systemically administered drugs to the human eye.

The nonsynonymous *ABCG2* single-nucleotide polymorphism (SNP) Q141K (c.421C > A), which affects the stability of the ABCG2 protein in the endoplasmic reticulum and enhances its susceptibility to proteosomal degradation (Furukawa et al., 2009), has been shown to lead to reduced transporter expression in different tissues (Kobayashi et al., 2005; Prasad et al., 2013; Tanaka et al., 2015). It has been reported that c.421AA carriers have an *in vivo* intestinal ABCG2 function approximately 23% of that in c.421CC subjects (Tanaka et al., 2015). The efficacy and the toxicity of diverse ABCG2 substrates, e.g., statin drugs, chemotherapy or allopurinol, has been found to be affected by the c.421C > A variant (Chen et al., 2019). Our previous data indicated that carriers of the c.421C > A SNP had diminished activity of ABCG2 at BBB, leading to increased susceptibility to ABCB1 inhibition (Bauer et al., 2016). It remains to be explored whether the c.421C > A SNP has an impact on ABCG2 function at the human BRB.

Positron emission tomography (PET) with radiolabelled transporter substrates allows to directly and non-invasively assess the influence of transporters at the BBB on drug distribution to the human brain (Bickel, 2005; Tournier et al., 2018). Next to measuring ABCB1 function at the BBB (Bauer et al., 2012; Bauer et al., 2015; Bauer et al., 2017), PET with the radiolabelled ABCB1 substrate (*R*)-[^11^C]verapamil has also been used to measure ABCB1 function at the human BRB (Bauer et al., 2017). PET with the dual ABCB1/ABCG2 substrate [^11^C]tariquidar (Bankstahl et al., 2013) with concurrent infusion of a high dose of unlabelled tariquidar to inhibit ABCB1 was successfully employed to reveal and measure for the first time the transport activity of ABCG2 at the human BBB (Bauer et al., 2016). In the present study, we aimed to extend the analysis of data from our previously published study in healthy volunteers (Bauer et al., 2016) to assess the impact of ABCB1 and ABCG2 and the *ABCG2* c.421C > A genotype on the distribution of [^11^C]tariquidar to the human retina.

## Methods

The study was registered with EUDRACT (number 2012-005796-14), approved by the Ethics Committee of the Medical University of Vienna, and conducted in accordance with the Declaration of Helsinki. The reported data are from an extended analysis of the previously published study of Bauer et al. (Bauer et al., 2016). Eleven out of 52 screened subjects were identified as carriers of the *ABCG2* c.421C > A SNP by means of probe-based polymerase chain reaction as previously described (Bauer et al., 2016). The sample management and the SNPgenotyping was performed at the MedUni Wien Biobank according to standard operating procedures (Haslacher et al., 2018). In total, five male subjects who were non-carriers (c.421CC) and five male subjects who were heterozygous carriers (c.421CA) of the *ABCG2* c.421C > A SNP and who were judged to be medication free and healthy based on the screening examinations, were enrolled into the [^11^C]tariquidar study arm. A summary table of the human subjects included is available in the Supplementary Table S1. The volunteers (mean age: 30 ± 9 years) underwent two consecutive 60 min PET scans on an Advance scanner (General Electric Medical Systems, Milwaukee, WI, United States) after intravenous injection of [^11^C]tariquidar (injected radioactivity amount: 388 ± 18 MBq). Serial arterial blood samples were drawn as previously described during the imaging sessions (Bauer et al., 2013). The second PET scan was performed with concurrent intravenous infusion of unlabelled tariquidar (AzaTrius Pharmaceuticals, Mumbai, India) to inhibit ABCB1 only and reveal ABCG2 function as previously described (Bauer et al., 2016). Tariquidar infusion was started 1 h before and continued until the end of the image acquisition (total infusion length: 120 ± 4 min). The total administered dose of unlabelled tariquidar was 5.8 ± 1.0 mg/kg body weight (mean subject weight: 80 ± 12 kg).

Region of interest (ROI) analysis was conducted for the retina on magnetic resonance (MR)-to-PET co-registered images based on individual T1-weighted MR images with PMOD software (version 3.6; PMOD Technologies Ltd., Zürich, Switzerland). Probabilistic atlas-based, whole brain grey matter (WBGM) data were already reported in (Bauer et al., 2016). A standard 2-tissue-4-rate constant compartmental (2T4K) model (see Supplementary Figure S1) was fitted to the time–activity curves (TACs) of [^11^C]tariquidar in the retina and in WBGM from 0 to 60 min after radiotracer injection (Bauer et al., 2013) using an arterial plasma input function which was not corrected for radiolabelled metabolites of [^11^C]tariquidar (due to the low percentage of radiolabelled metabolites in plasma). Modelling outcome parameters were the radiotracer transfer rate constants across the BRB and BBB between plasma and the first tissue compartment (influx rate constant *K*_1_ and efflux rate constant *k*_2_) as well as between the first and second tissue compartments (influx rate constant *k*_3_ and efflux rate constant *k*_4_) (see Supplementary Figure S1). The fractional arterial blood volume in tissue (*V*_b_) was included as a fitting parameter. Logan graphical analysis was used to estimate the total volume of distribution (*V*_T_) in a model-independent manner (Logan et al., 1990). *V*_T_ equals the tissue-to-plasma concentration ratio at steady state. All data are given as arithmetic mean ± standard deviation (SD). Differences in the outcome parameters between scan 1 and 2 were tested using the Wilcoxon signed rank test and between groups using the Mann-Whitney test (Statistica 6.1, StatSoft, Tulsa, OK, United States). To assess correlations, the Spearman rank correlation coefficient *r*_*s*_ was calculated. A *p* value of less than 0.05 was considered statistically significant.

We additionally performed an extended data analysis of a (*R*)-[^11^C]verapamil PET data set previously published by our group (Wagner et al., 2009) in which 5 healthy volunteers underwent two consecutive (*R*)-[^11^C]verapamil PET scans before and after administration of tariquidar at a dose which only partially inhibits ABCB1 function at the BBB (2 mg/kg) in order to investigate the effect of this tariquidar dose on ABCB1 function at the BRB. The methods used are described in the Supplementary Material.

## Results

During the experiments only mild or moderate adverse events were recorded and are listed in reference (Bauer et al., 2016).

**FIGURE 1:**
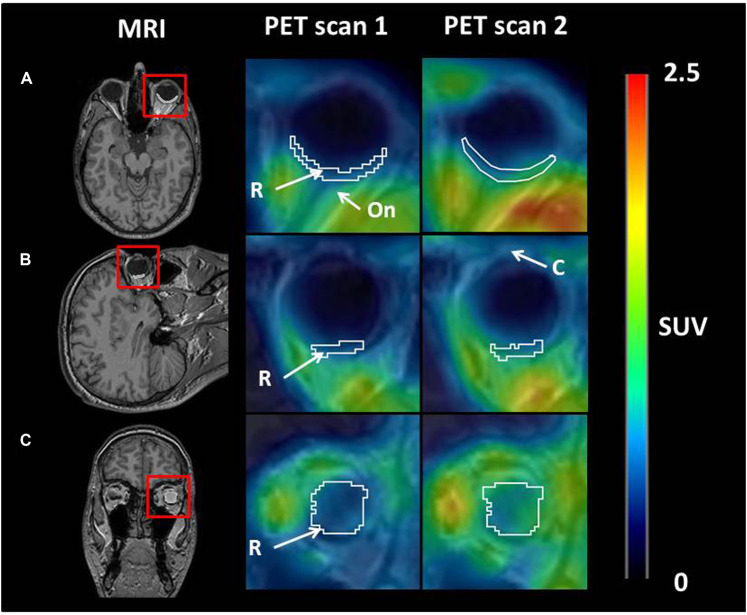
Axial **(A)**, sagittal **(B)**, and coronal **(C)** planes of representative MR and PET average images (0–60 min) at baseline (scan 1) and during ABCB1 inhibition (scan 2) in one c.421CA carrier. Red rectangles on MR images indicate magnified area on PET images. A representative region of interest for retina (white contour) is shown. Anatomical structures are labelled with arrows: R, retina; On, optical nerve; C, cornea. Radiation scale is expressed as standardized uptake value (SUV) and set from 0 to 2.5.

**FIGURE 2:**
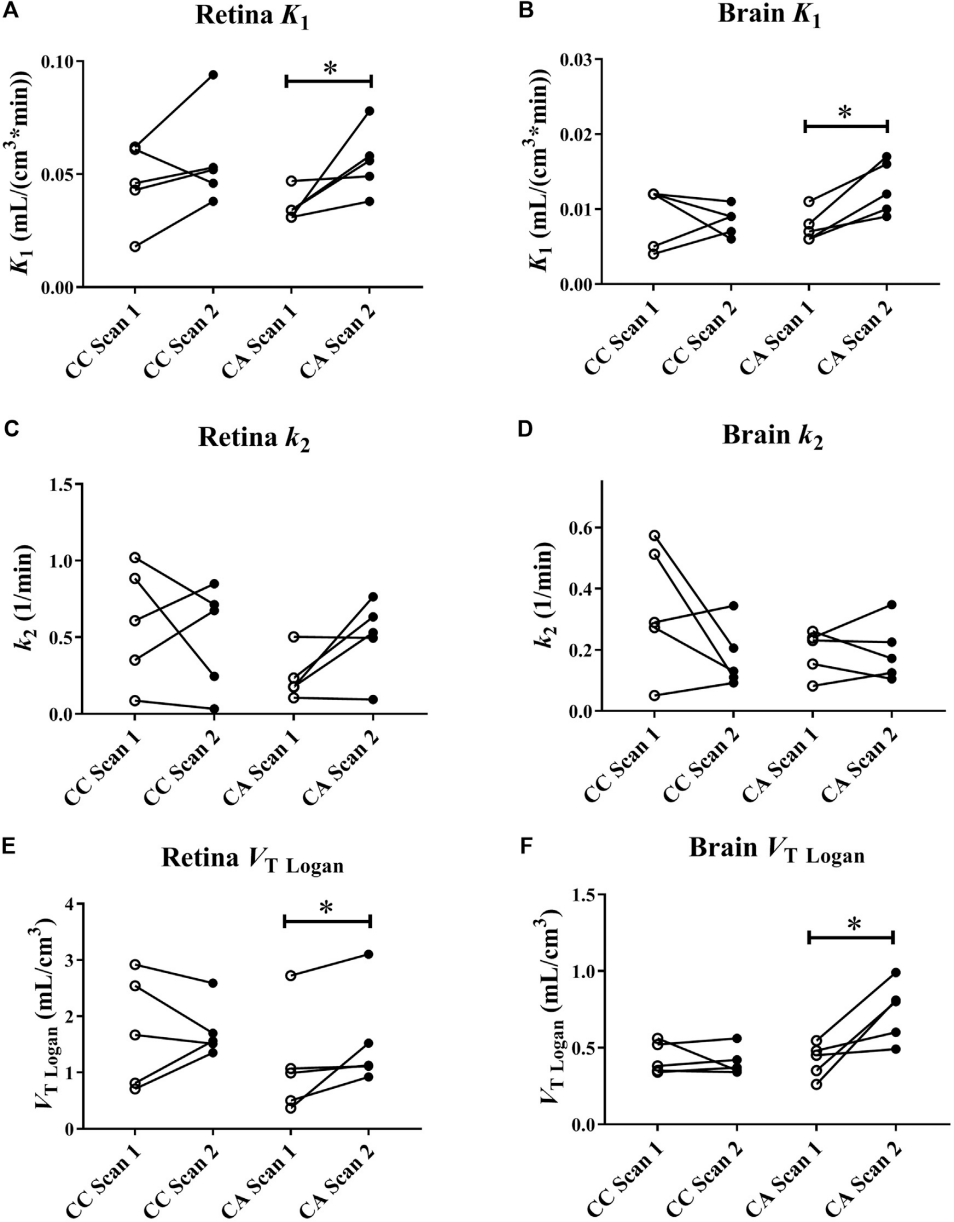
[^11^C]tariquidar modelling outcome parameters (*K*_1_ and *k*_2_ estimated from 2T4K model and *V*_T_ estimated with Logan graphical analysis) for retina **(A, C, and E)** and whole brain grey matter **(B, D, and F)** in c.421CC (CC) and c.421CA (CA) subjects for baseline scan (scan 1) and scan during ABCB1 inhibition (scan 2). Brain data are taken from Bauer et al. (Bauer et al., 2016). *p < 0.05, Wilcoxon signed rank test.

Figure 1 shows representative [^11^C]tariquidar PET images for scans without and with ABCB1 inhibition of one c.421CA carrier with the outlined retina ROI. Mean modelling outcome parameters for the retina and the brain for the two PET scans in c.421CC and c.421CA carriers are given in Table 1. In Figure 2, selected modelling outcome parameters for the baseline scan and the scan with ABCB1 inhibition for the retina and the brain in individual c.421CC and c.421CA subjects are displayed.

**TABLE 1:** [^11^C]Tariquidar modelling outcome parameters for the retina and whole brain grey matter in c.421CC and c.421CA subjects for the baseline scan and the scan during ABCB1 inhibition with unlabelled tariquidar.

Region of interest	Group	*K*_1_ (mL/(cm^3.^min))	*k*_2_ (1/min)	*k*_3_ (1/min)	*k*_4_ (1/min)	*V*_T Logan_ (ml/cm^3^)	*V*_b_
Retina	c.421CC baseline	0.046 ± 0.018 (28)	0.590 ± 0.382 (138)	0.593 ± 0.554 (134)	0.037 ± 0.050 (158)	1.730 ± 0.995 (53)	0.009 ± 0.002 (67)
	c.421CC ABCB1 inhibition	0.057 ± 0.022 (32)	0.503 ± 0.347 (180)	0.294 ± 0.099 (501)	0.151 ± 0.294 (119)	1.742 ± 0.490 (7)	0.010 ± 0.006 (31)
	c.421CA baseline	0.035 ± 0.007 (29)	0.239 ± 0.154 (95)	0.150 ± 0.091 (95)	0.041 ± 0.029 (75)	1.130 ± 0.939 (10)	0.010 ± 0.003 (27)
	c.421CA ABCB1 inhibition	0.056 ± 0.015 (45)*	0.503 ± 0.252 (118)	0.390 ± 0.236 (63)*	0.113 ± 0.163 (54)	1.556 ± 0.890 (19)*	0.011 ± 0.007 (81)
Whole brain grey matter	c.421CC baseline	0.009 ± 0.004 (26)	0.340 ± 0.209 (57)	0.152 ± 0.037 (34)	0.012 ± 0.006 (23)	0.430 ± 0.102 (8)	0.047 ± 0.005 (8)
	c.421CC ABCB1 inhibition	0.008 ± 0.002 (16)	0.176 ± 0.103 (46)	0.119 ± 0.050 (32)	0.014 ± 0.005 (25)	0.408 ± 0.090 (7)	0.046 ± 0.006 (8)
	c.421CA baseline	0.008 ± 0.002 (32)	0.193 ± 0.074 (70)	0.116 ± 0.055 (38)	0.010 ± 0.001 (41)	0.417 ± 0.112 (13)	0.055 ± 0.008 (9)
	c.421CA ABCB1 inhibition	0.013 ± 0.004 (13)*	0.195 ± 0.097 (34)	0.140 ± 0.040 (22)	0.015 ± 0.006 (18)	0.738 ± 0.196 (8)*	0.047 ± 0.008 (10)*

Values are reported as arithmetic mean ± standard deviation. The value in parentheses represents the precision of the parameter estimates (expressed as their mean standard error in percent). *K*_1_ (mL/(cm^3^.min)), rate constant for radiotracer transfer from plasma into the first tissue compartment; *k*_2_ (1/min), rate constant for radiotracer transfer from the first tissue compartment into plasma; *k*_3_ (1/min), rate constant for radiotracer transfer from the first tissue compartment into the second tissue compartment; *k*_4_ (1/min), rate constant for radiotracer transfer from the second tissue compartment into the first tissue compartment; *V*_T Logan_ (ml/cm^3^), total volume of distribution estimated with Logan graphical analysis; *V*_b_, fractional arterial blood volume in the eyes/brain. *p < 0.05 for comparison with baseline scan using the Wilcoxon signed rank test.

For the baseline scans, *K*_1_ and *V*_T_ values of [^11^C]tariquidar were 4 to 5-fold higher for the retina than for WBGM. Baseline distribution of [^11^C]tariquidar to the retina as well as to WBGM did not significantly differ between c.421CC and c.421CA subjects. In response to ABCB1 inhibition with unlabelled tariquidar, *ABCG2* c.421C > A genotype carriers showed significant increases as compared to the baseline scan in retinal radiotracer influx (*K*_1_: +62 ± 57%, *p* = 0.043), and *V*_T__Logan_ (+86 ± 131%, *p* = 0.043) (Figure 2). No significant changes in any of the modelling outcome parameters were observed in subjects with the c.421CC genotype (Figure 2). The BRB findings were in good agreement with those for the BBB, for which *K*_1_ (+72 ± 35%, *p* = 0.043) and *V*_T Logan_ (+91 ± 82%, *p* = 0.043) were also significantly and with a similar magnitude increased following ABCB1 inhibition in c.421CA subjects only. In c.421CA subjects, there was a trend towards a positive correlation between the percentage change in *V*_T Logan_ in the retina and in the brain in scan 2 relative to scan 1 (*r*_s_ = 0.8, *p* = 0.133, not shown). In contrast to the BRB, *k*_3_ values were not significantly increased in the brain of c.421CA subjects following ABCB1 inhibition (Table 1).

The mean modelling outcome parameters for the retina and the brain for the (*R*)-[^11^C]verapamil PET scans without and with ABCB1 inhibition with a lower dose of unlabelled tariquidar (2 mg/kg) are reported in the Supplementary Table S2. In contrast to [^11^C]tariquidar, baseline *K*_1_ and *V*_T_ values were for (*R*)-[^11^C]verapamil comparable for the retina and for the brain. Following ABCB1 inhibition, *K*_1_ and *V*_T_ values of (*R*)-[^11^C]verapamil were significantly increased as compared with the baseline scan, both for the retina and the brain (*K*_1_, retina: +97 ± 100%, *p* = 0.043; *K*_1_, WBGM: +49 ± 36%, *p* = 0.043; *V*_T Logan_, retina: +43 ± 30%, *p* = 0.043; *V*_T Logan_, WBGM: +24 ± 15%, *p* = 0.043).

## Discussion

In this study, we used PET imaging to assess the functional impact of the two efflux transporters ABCB1 and ABCG2 at the BRB on controlling the distribution of a model ABCB1/ABCG2 substrate ([^11^C]tariquidar) to the human retina. Tariquidar is a non-marketed, third-generation ABCB1 inhibitor, which was originally developed to overcome multidrug resistance in cancer patients (Fox and Bates, 2007) and which has been re-purposed to inhibit ABCB1 at the BBB in an experimental setting (Bauer et al., 2012; Bauer et al., 2015; Bauer et al., 2017). Interestingly, non-clinical data indicated that tariquidar highly accumulates in the eye, which has been attributed to binding to melanin (INVESTIGATOR BROCHURE Tariquidar, 2007), which is abundantly expressed in RPE cells (Rimpelä et al., 2018). At tracer doses [^11^C]tariquidar is transported by both ABCB1 and ABCG2 and has been employed as a PET tracer to measure the activity of ABCB1 and ABCG2 at the BBB (Bankstahl et al., 2013; Bauer et al., 2016). In the present study, we extend for the first time previous *ex vivo* investigations on the impact of ABCB1 on the distribution of diverse model ABCB1 substrates to the mouse and rat eye (Hosoya et al., 2010; Toda et al., 2011; Fujii et al., 2014; Chapy et al., 2016) to a dual ABCB1/ABCG2 substrate examined *in vivo* in humans. We further investigated the effect of the *ABCG2* c.421C > A genotype on the retinal distribution of [^11^C]tariquidar. We performed two consecutive [^11^C]tariquidar PET scans in five subjects who were carriers of the fully functioning *ABCG2* allele (c.421CC) and in five subjects with the c.421CA genotype which presumably results in reduced ABCG2 abundance and function. Following the baseline PET scan, a second scan was performed with a concurrent high dose infusion of unlabelled tariquidar to achieve significant ABCB1 inhibition and thereby specifically reveal ABCG2 function at the human BRB (Bauer et al., 2015; Bauer et al., 2016). Data obtained with [^11^C]tariquidar for the BRB were compared with data previously obtained with [^11^C]tariquidar for the BBB (Bauer et al., 2015; Bauer et al., 2016) and with data obtained with the ABCB1-selective substrate radiotracer (*R*)-[^11^C]verapamil.

One first important finding of our study was that [^11^C]tariquidar distribution across the BRB to the retina (*K*_1_, *V*_T_) was considerably higher (4 to 5 fold) than its distribution across the BBB to the brain, regardless of the genotype and ABCB1 inhibition condition (see Table 1). This is largely consistent with the results from studies in rodents, which revealed a higher distribution of prototypical ABCB1 substrates (e.g., verapamil, quinidine and digoxin) to the retina than to the brain (Hosoya et al., 2010; Toda et al., 2011; Fujii et al., 2014; Chapy et al., 2016). This has been interpreted in a way that ABCB1-mediated efflux is quantitatively less important at the rodent BRB than at the rodent BBB, while passive transcellular permeability appears to be similar at the BRB and BBB for lipophilic compounds (Hosoya et al., 2010). However, our results obtained with [^11^C]tariquidar differed from those obtained with (*R*)-[^11^C]verapamil in humans, for which *K*_1_ and *V*_T_ values were comparable for the retina and the brain (see Supplementary Table S2 and reference (Bauer et al., 2017). This may point to species differences between rodents and humans with regards to the retinal distribution of verapamil, which may be possibly related to the presence of an unidentified uptake transporter which mediates verapamil uptake across the BRB (Hosoya et al., 2010; Kubo et al., 2013; Chapy et al., 2016).

A second important finding of our study was that the BRB efflux transport function in c.421CC carriers remained unaltered during ABCB1 inhibition which confirms for the first time the presence of functional ABCG2 at the human BRB. This suggests that a similar functional redundancy between ABCB1 and ABCG2 as described for the rodent and human BBB (Kodaira et al., 2010; Bauer et al., 2016) exists at the human BRB in controlling the retinal distribution of dual ABCB1/ABCG2 substrates.

Our results showed that carriers of the c.421C > A SNP had significant increases in retinal distribution of [^11^C]tariquidar following ABCB1 inhibition, while c.421CC subjects did not. This supports that the investigated *ABCG2* SNP decreased the function of ABCG2 at the BRB, just as previously reported for the BBB (Bauer et al., 2016). This may suggest that SNP carriers may be more susceptible to transporter-mediated DDIs at the BRB than non-carriers because the functional redundancy between ABCB1 and ABCG2 is compromised.

The percentage increase in retinal distribution of [^11^C]tariquidar in c.421CA subjects following complete ABCB1 inhibition was of similar magnitude as the percentage increase in its brain distribution (Table 1). This again contrasted with results obtained with (*R*)-[^11^C]verapamil for which the increase in brain uptake was considerably higher than the increase in retinal uptake (3.8- *vs.* 1.5-fold increase in *V*_T_) following complete ABCB1 inhibition by employing the same tariquidar infusion protocol as in the present study (Bauer et al., 2017). This could mean that the functional impact of ABCG2 is comparable at the human BRB and BBB, while the functional impact of ABCB1 is lower at the human BRB than at the human BBB. However, the presence of an uptake transport system for verapamil at the BRB, which effect was revealed only when ABC efflux transport was abolished, complicates the comparison of the response to ABCB1 inhibition between (*R*)-[^11^C]verapamil and [^11^C]tariquidar (Chapy et al., 2016).

The percentage increase in (*R*)-[^11^C]verapamil *K*_1_ and *V*_T_ values at the BRB following administration of a tariquidar dose (2 mg/kg) which only partially blocks ABCB1 at the human BBB (Wagner et al., 2009; Bauer et al., 2015) (see Supplementary Table S2) was comparable to the percentage increase following administration of a tariquidar dose which almost completely blocks ABCB1 at the human BBB (see reference (Bauer et al., 2017). This indicated that that the low dose of tariquidar (2 mg/kg) may have already led to complete inhibition of ABCB1 at the BRB. This may suggest that ABCB1 at the human BBB is less susceptible to inhibition than ABCB1 at the human BRB. Consequently, lower doses of ABCB1 inhibitors may lead to significant changes in retinal distribution of ABCB1 substrates than those needed for inhibition of ABCB1 at the BBB. This could suggest that there is an overall higher risk for ABCB1-mediated DDIs to occur at the BRB than at the BBB. These findings are in line with those of a study in mice which revealed the need of only half of the dose of an ABCB1 inhibitor (elacridar) to achieve full ABCB1 inhibition at the BRB than for the BBB (Chapy et al., 2016). The authors linked this finding to lower activity of ABCB1 at the BRB as compared to the BBB.

On one hand, transporter-mediated DDIs at the BRB due to concomitant treatment with drugs which inhibit ABCB1 and/or ABCB2 could contribute to enhanced ocular and in particular retinal toxicity of drugs that normally penetrate poorly into the eye, such as imatinib (Ho et al., 2013), other tyrosine kinase inhibitors (Williamson and Reddy, 2021), ciprofloxacin (Ramirez et al., 2011), tamoxifen (Griffin and Garnick, 1981; Noureddin et al., 1999; Grzybowski et al., 2015) and methotrexate (Balachandran et al., 2002; Iqbal et al., 2005; Sbeity et al., 2006; Sharma and Sharma, 2011; Grzybowski et al., 2015) (see Supplementary Table S3). On the other hand, DDIs could generate therapeutic benefits by enhancing the ocular penetration of systemic treatments for retinal disorders struggling to cross the BRB, which represents so far a major challenge for ocular drug delivery (Jordán and Ruíz-Moreno, 2013; Agrahari et al., 2016; Pascual-Pasto et al., 2017; Kim and Woo, 2021). This may be the useful for an improved treatment of diseases, such as neovascular age-related macular degeneration, diabetic retinopathy, and retinal vascular disorders, which are the leading causes of vision deterioration in most developed countries (Bickel, 2005). There is therefore a need for methodology, such as PET imaging, to measure the ocular disposition of drugs in humans.

Just as for the BBB (Kalvass et al., 2013; Bauer et al., 2016), the likelihood of clinically relevant DDIs at the human BRB for dual ABCB1/ABCG2 substrate drugs is likely to be low if ABCB1 and ABCG2 function is preserved, since both transporters possess mutual functional redundancy. Nevertheless, some physiological or pathological conditions have been associated with a reduction in the abundance of ABCB1 and ABCG2 at the BBB as for instance healthy ageing or Alzheimer’s disease (Kannan et al., 2017; Storelli et al., 2020), which may raise the risk for ABCB1-mediated DDIs at the BBB and central side effects in the elderly (Bauer et al., 2017; Bauer et al., 2021). Consequently, a clinically achievable degree of ABCB1 inhibition in the brain and retina could be sufficient to lead to significantly higher tissue exposure in *ABCG2* c.421C > A genotype carriers and thereby increase the risk of side effects for ABCB1/ABCG2 substrate drugs with a narrow therapeutic index. The expression of the c.421C > A SNP, which is one of the most common reduced-function variants of ABCG2, is highly dependent on ethnicity. The heterozygous (c.421CA) and homozygous (c.421AA) variants occur with a frequency of 40–45% and 8–12%, respectively, in the East Asian population (Chinese, Japanese, Korean), whereas c.421CA and c.421AA carriers are rare in Caucasians (frequency of 17 and 1%, respectively) and even rarer in Africans (combined frequency of 1.3%) (Lai et al., 2012; Li and Barton, 2018; Chen et al., 2019). The enhanced risk for transporter-mediated DDIs under certain pathological conditions and in SNP carriers may play a clinical role for subjects undergoing treatment with potentially retinotoxic drugs such as tamoxifen or methotrexate (Grzybowski et al., 2015). Selected ABCG2 substrates from references (Mao and Unadkat, 2015; Fohner et al., 2017) with potential retinotoxicity or ocular therapeutic applications are listed in Supplementary Table S3.

One limitation of our study was that we did not measure effective retinochoroidal blood flow (RCBF) in study participants. Retinal uptake of [^11^C]tariquidar and (*R*)*-*[^11^C]verapamil may partly depend on RCBF and may thus need correction for RCBF to specifically reveal the function of ABCB1 and ABCG2 at the BRB. While previous data suggested that administration of a pharmacological dose of tariquidar does not affect cerebral blood flow in humans (Kreisl et al., 2010), its effect on RCBF currently remains unknown. The choroidal circulation provides blood supply for the outer retina and particularly the photo-receptors while the central retinal artery irrigates the inner retinal layers (Vaghefi & Pontre, 2016). This dual source of retinal blood supply complicates the estimation of the impact of RCBF variation on [^11^C]tariquidar and (*R*)*-*[^11^C]verapamil distribution to the retina in particular in case of inhomogeneous expression of ABCG2/ABCB1 between the inner and outer BRB. Hence it is not possible to exclude that tariquidar-induced increases in RCBF could have at least partly contributed to the observed increases in retinal [^11^C]tariquidar and (*R*)*-*[^11^C]verapamil uptake.

Another limitation of the study is that we could only include heterozygous (c.421CA) but no homozygous carriers (c.421AA) of the *ABCG2* c.421C > A SNP owing to the rarity of this polymorphism in the Caucasian population.

In conclusion, our study highlights the potential utility of PET imaging to non-invasively assess ocular disposition of drugs in humans. We provide the first evidence that, in analogy to the BBB, ABCB1 and ABCG2 may together limit at the BRB the distribution of systemically administered ABCB1/ABCG2 substrate drugs to the human retina. Carriers of the c.421C > A SNP may be more susceptible to transporter-mediated DDIs at the BRB than non-carriers. This may play a role for subjects undergoing treatment with potentially retinotoxic drugs such as tamoxifen or methotrexate.

## Data Availability

The original contributions presented in the study are included in the article/Supplementary Material, further inquiries can be directed to the corresponding author.
