# Supplementary material for: ABCB1 and ABCG2 Together Limit the Distribution of ABCB1/ABCG2 Substrates to the Human Retina and the ABCG2 Single Nucleotide Polymorphism Q141K (c.421C> A) May Lead to Increased Drug Exposure
Source: Front Pharmacol. 2021 Jun 16;12:698966. doi: 10.3389/fphar.2021.698966 (PMC8242189; doi:10.3389/fphar.2021.698966)
Supplement: Supplementary file 1 [file DataSheet1.docx]

Supplementary Material

# Supplementary: Methods for additional analysis of (*R*)-[^11^C]verapamil PET data

# In order to investigate the effect of partial inhibition of ABCB1 function at the BRB, we performed an additional and extended data analysis of another study of our group ([1](#_ENREF_1)). Datasets of five healthy volunteers who underwent (*R*)-[^11^C]verapamil PET scans before and approximately 3 hours after intravenous administration of tariquidar (2 mg/kg of body weight) were analyzed. More details on the study can be found in the original article ([1](#_ENREF_1)). In brief, a standard 2-tissue-4-rate-constant compartmental (2T4K) model was fitted to the (*R*)-[^11^C]verapamil TACs in the retina and in WBGM from 0 to 40 minutes after radiotracer injection using a metabolite-corrected arterial plasma input function. The 2T4K model failed to provide reliable estimates of *k*_3_ and *k*_4_ in the retina, so that only *K*_1_ and *k*_2_ values and volume of distribution (*V*_T_) values estimated with Logan graphical analysis are reported. All data are given as arithmetic mean ± standard deviation (SD). Differences in the outcome parameters of scans 1 and 2 were tested using the Wilcoxon signed rank test (Statistica 6.1, StatSoft, Tulsa, OK, USA). A *p* value of less than 0.05 was considered statistically significant.

**Supplementary Table S1**: Summary table of the human subjects included in the [^11^C]tariquidar study.

| **Subject code** | **Sex** | **Age** (years) | **Weight** (kg) | ***ABCB1* SNP** | | | ***ABCG2* SNP** |
| --- | --- | --- | --- | --- | --- | --- | --- |
|  |  |  |  | G2677T | C3435T | C1236T | Q141K |
|  |  |  |  | **rs2032582** | **rs1045642** | **rs1128503** | **rs2231142** |
| **p01** | male | 23 | 72 | **[G];[T]** | **[C];[T]** | **[C];[T]** | **[C];[C]** |
| **p04** | male | 33 | 71 | **[G];[G]** | **[C];[C]** | **[C];[C]** | **[C];[C]** |
| **p05** | male | 34 | 87 | **[G];[G]** | **[C];[C]** | **[C];[C]** | **[C];[C]** |
| **p06** | male | 35 | 90 | **[G];[G]** | **[C];[C]** | **[C];[C]** | **[C];[C]** |
| **p19** | male | 24 | 59 | **n.c.** | **[T];[T]** | **[C];[C]** | **[C];[C]** |
| **p02** | **male** | **51** | **76** | **[G];[T]** | **[C];[T]** | **[C];[T]** | **[C];[A]** |
| **p03** | **male** | **22** | **79** | **[G];[T]** | **[C];[T]** | **[C];[T]** | **[C];[A]** |
| **p20** | **male** | **28** | **98** | **[T];[T]** | **[T];[T]** | **[T];[T]** | **[C];[A]** |
| **p21** | **male** | **25** | **86** | **[G];[T]** | **[T];[T]** | **[C];[T]** | **[C];[A]** |
| **p28** | **male** | **23** | **75** | **[G];[G]** | **[C];[C]** | **[C];[C]** | **[C];[A]** |

SNP = single-nucleotide polymorphism; n.c. = not conclusive

#
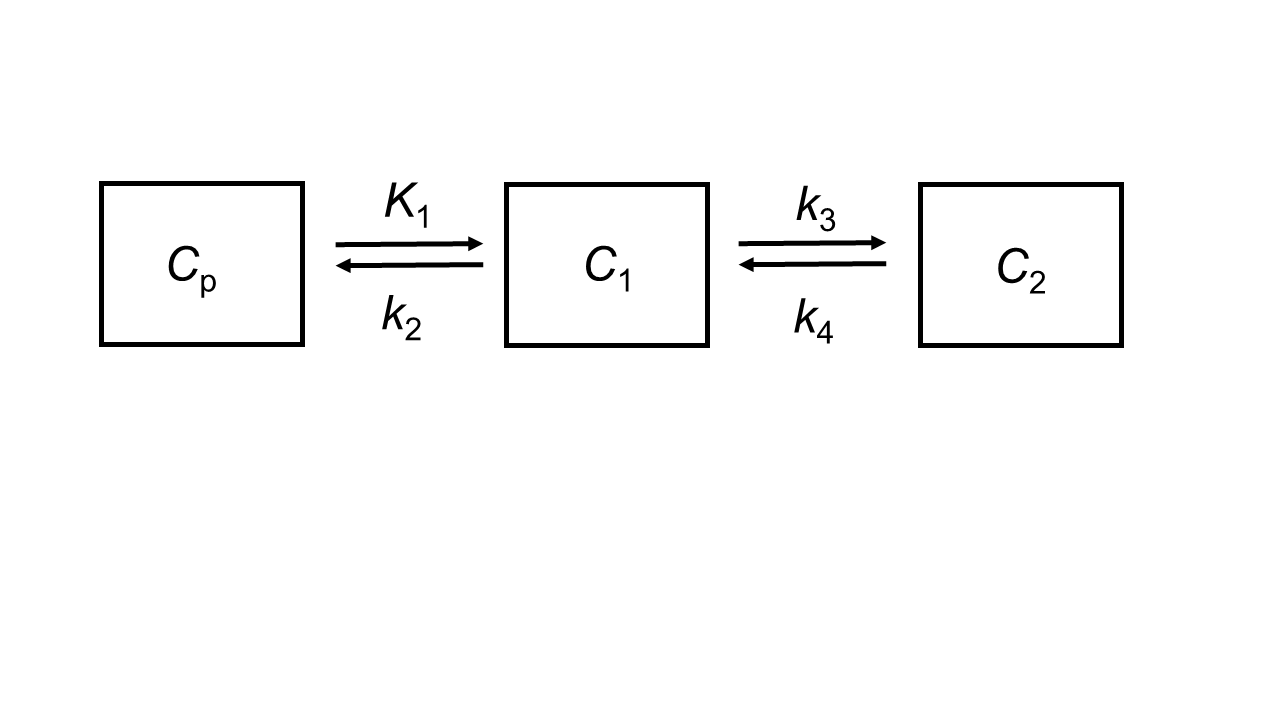


# Supplementary Figure S1. Diagram of the 2-tissue-4-rate constant compartmental (2T4K) model used for the kinetic modelling of [^11^C]tariquidar and (*R*)-[^11^C]verapamil PET data for the retina and the brain. *C*_p_ is the concentration of radiotracer in arterial plasma and *C*_1_ and *C*_2_ denote the radiotracer concentrations in the first and second tissue compartments of the retina or brain. *K*_1_ (mL/(cm^3^.min)) is the rate constant for radiotracer transfer from plasma into the first tissue compartment, *k*_2_ (1/min) is the rate constant for radiotracer transfer from the first tissue compartment into plasma, *k*_3_ (1/min) is the rate constant for radiotracer transfer from the first tissue compartment into the second tissue compartment and *k*_4_ (1/min) is the rate constant for radiotracer transfer from the second tissue compartment into the first tissue compartment.

**Supplementary Table S2:** (*R*)-[^11^C]verapamil modelling outcome parameters for the retina and whole brain grey matter for the baseline scan and the scan during ABCB1 inhibition with unlabelled tariquidar (2 mg/kg body weight)

| **Region of interest** | **Group** | ***K*_1_ (mL/(cm^3.^min))** | ***k*_2_ (1/min)** | ***V*_T Logan_ (mL/cm^3^)** |
| --- | --- | --- | --- | --- |
| Retina | **Baseline** | 0.035±0.011 (39) | 0.196±0.217 (929) | 0.71±0.231 (10) |
|  | **ABCB1 inhibition** | 0.061±0.014 (18)* | 0.335±0.252 (101) | 0.97±0.22 (8)* |
| Whole brain grey matter | **Baseline** | 0.034±0.009 (15) | 0.091±0.007 (111) | 0.64±0.12 (1) |
|  | **ABCB1 inhibition** | 0.049±0.009 (9)* | 0.128±0.070 (59) | 0.79±0.07 (1)* |

Values are reported as mean ± standard deviation. The value in parentheses represents the precision of the parameter estimates (expressed as their mean standard error in percent). *K*_1_ (mL/(cm^3^.min)), rate constant for radiotracer transfer from plasma into the first tissue compartment; *k*_2_ (1/min), rate constant for radiotracer transfer from the first tissue compartment into plasma; *V*_T Logan_ (mL/cm^3^), total volume of distribution estimated with Logan graphical analysis. Brain data are taken from Wagner et al. ([1](#_ENREF_1)). * *p* < 0.05 for comparison with baseline scan using the Wilcoxon signed rank test.

**Supplementary Table S3:** List of selected ABCG2 substrates from references ([2](#_ENREF_2), [3](#_ENREF_3)) with potential retinotoxicity, ocular therapeutic applications or without evidence of retinotoxicity

| ABCG2 substrates with risk of retinotoxicity | Ref. | ABCG2 substrates with potential application in ophtalmology | Potential ophtalmologic applications | Ref. | ABCG2 substrates without evidence of retinotoxicity |
| --- | --- | --- | --- | --- | --- |
| 5-fluorouracil | ([4](#_ENREF_4)) | atorvastatin | DRP, CNV | ([5-7](#_ENREF_5)) | abacavir |
| ciprofloxacin | ([8](#_ENREF_8), [9](#_ENREF_9)) | daunorubicin | PVR | ([10](#_ENREF_10)) | doxorubicin |
| daunorubicin | ([11](#_ENREF_11)) | fluvastatin | DRP, RNP | ([12](#_ENREF_12), [13](#_ENREF_13)) | epirubicin |
| efavirenz | ([14-16](#_ENREF_14)) | folic acid | AMD, DRP | ([17-19](#_ENREF_17)) | erlotinib |
| imatinib | ([20-26](#_ENREF_20)) | gefitinib | AMD, DRP | ([27](#_ENREF_27)) | erythromycin |
| ganciclovir | ([28](#_ENREF_28)) | glyburide | DRP | ([29](#_ENREF_29)) | etoposide |
| methotrexate | ([9](#_ENREF_9), [30-33](#_ENREF_30)) | pitavastatin | RNP | ([34](#_ENREF_34)) | irinotecan |
| nilotinib | ([23](#_ENREF_23), [35](#_ENREF_35)) | pravastatin | DRP, RNP | ([7](#_ENREF_7), [13](#_ENREF_13), [36](#_ENREF_36)) | lamivudine |
| tamoxifen | ([9](#_ENREF_9), [37](#_ENREF_37), [38](#_ENREF_38)) | simvastatin | DRP | ([5-7](#_ENREF_5)) | pantoprazole |
| folic acid (deficiency) | ([9](#_ENREF_9), [31](#_ENREF_31)) | sulfasalazine | PTR | ([39](#_ENREF_39)) | rosuvastatin |
|  |  | topotecan | Retinoblastoma | ([40](#_ENREF_40)) | sorafenib |
|  |  |  |  |  | sunitinib |
|  |  |  |  |  | zidovudine |

DRP = diabetic retinopathy; RNP = retinal neuroprotection; CNV = choroidal neovascularisation; AMD = age-related macular degeneration; PVR = proliferative vitreoretinopathy; PTR = prevention tamoxifen retinotoxicity

References

1. Wagner CC, Bauer M, Karch R, Feurstein T, Kopp S, Chiba P, et al. A pilot study to assess the efficacy of tariquidar to inhibit P-glycoprotein at the human blood-brain barrier with (R)-11C-verapamil and PET. J Nucl Med. 2009;50(12):1954-61. Epub 2009/11/17.

2. Mao Q, Unadkat JD. Role of the breast cancer resistance protein (BCRP/ABCG2) in drug transport--an update. AAPS J. 2015;17(1):65-82. Epub 2014/09/23.

3. Fohner AE, Brackman DJ, Giacomini KM, Altman RB, Klein TE. PharmGKB summary: very important pharmacogene information for ABCG2. Pharmacogenet Genomics. 2017;27(11):420-7. Epub 2017/09/01.

4. Raina AJ, Gilbar PJ, Grewal GD, Holcombe DJ. Optic neuritis induced by 5-fluorouracil chemotherapy: Case report and review of the literature. J Oncol Pharm Pract. 2020;26(2):511-6. Epub 2019/11/19.

5. Ioannidou E, Tseriotis VS, Tziomalos K. Role of lipid-lowering agents in the management of diabetic retinopathy. World J Diabetes. 2017;8(1):1-6. Epub 2017/02/01.

6. Mozetic V, Pacheco RL, Latorraca COC, Riera R. Statins and/or fibrates for diabetic retinopathy: a systematic review and meta-analysis. Diabetol Metab Syndr. 2019;11:92. Epub 2019/11/14.

7. Al-Janabi A, Lightman S, Tomkins-Netzer O. 'Statins in retinal disease'. Eye (Lond). 2018;32(5):981-91. Epub 2018/03/21.

8. Etminan M, Forooghian F, Brophy JM, Bird ST, Maberley D. Oral fluoroquinolones and the risk of retinal detachment. JAMA. 2012;307(13):1414-9. Epub 2012/04/05.

9. Grzybowski A, Zulsdorff M, Wilhelm H, Tonagel F. Toxic optic neuropathies: an updated review. Acta Ophthalmol. 2015;93(5):402-10. Epub 2014/08/28.

10. Kumar A, Nainiwal S, Choudhary I, Tewari HK, Verma LK. Role of daunorubicin in inhibiting proliferative vitreoretinopathy after retinal detachment surgery. Clin Exp Ophthalmol. 2002;30(5):348-51. Epub 2002/09/06.

11. Mathew DJ, Arthur A, John SS. Presumed Chemotherapy-Induced Optic Neuropathy and Maculopathy: A Case Report. Open Ophthalmol J. 2017;11:298-304. Epub 2018/01/05.

12. Bartoli M, Al-Shabrawey M, Labazi M, Behzadian MA, Istanboli M, El-Remessy AB, et al. HMG-CoA reductase inhibitors (statin) prevents retinal neovascularization in a model of oxygen-induced retinopathy. Invest Ophthalmol Vis Sci. 2009;50(10):4934-40. Epub 2008/12/23.

13. Fernandez-Navarro J, Aldea P, de Hoz R, Salazar JJ, Ramirez AI, Rojas B, et al. Neuroprotective Effects of Low-Dose Statins in the Retinal Ultrastructure of Hypercholesterolemic Rabbits. PLoS One. 2016;11(5):e0154800. Epub 2016/05/06.

14. Curi AL, Freeman G, Kapembwa M, Pavesio C. Retinal toxicity due to Efavirenz. Eye (Lond). 2001;15(Pt 2):246-8. Epub 2001/05/08.

15. Sen P, Sudharshan S, Banerjee A, Dhami A. Clinical and electrophysiological characteristics of Efavirenz-induced macular toxicity. GMS Ophthalmol Cases. 2020;10:Doc08. Epub 2020/04/10.

16. Vaz Pereira C, Franco M, Guerra Pinto R, Lino S, Maltez F, Barrão S, et al. Bull’s Eye Maculopathy in a patient treated with efavirenz. Oftalmologia. 2014;39(1):69-72.

17. Christen WG, Glynn RJ, Chew EY, Albert CM, Manson JE. Folic acid, pyridoxine, and cyanocobalamin combination treatment and age-related macular degeneration in women: the Women's Antioxidant and Folic Acid Cardiovascular Study. Arch Intern Med. 2009;169(4):335-41. Epub 2009/02/25.

18. Ola MS, Nawaz MI, Khan HA, Alhomida AS. Neurodegeneration and neuroprotection in diabetic retinopathy. Int J Mol Sci. 2013;14(2):2559-72. Epub 2013/01/30.

19. Mansour SE, Browning DJ, Wong K, Flynn HW, Jr., Bhavsar AR. The Evolving Treatment of Diabetic Retinopathy. Clin Ophthalmol. 2020;14:653-78. Epub 2020/03/19.

20. Govind Babu K, Attili VS, Bapsy PP, Anupama G. Imatinib-induced optic neuritis in a patient of chronic myeloid leukemia. Int Ophthalmol. 2007;27(1):43-4. Epub 2007/04/06.

21. Bajel A, Bassili S, Seymour JF. Safe treatment of a patient with CML using dasatinib after prior retinal oedema due to imatinib. Leuk Res. 2008;32(11):1789-90. Epub 2008/05/09.

22. Kusumi E, Arakawa A, Kami M, Kato D, Yuji K, Kishi Y, et al. Visual disturbance due to retinal edema as a complication of imatinib. Leukemia. 2004;18(6):1138-9. Epub 2004/04/16.

23. Fu C, Gombos DS, Lee J, George GC, Hess K, Whyte A, et al. Ocular toxicities associated with targeted anticancer agents: an analysis of clinical data with management suggestions. Oncotarget. 2017;8(35):58709-27. Epub 2017/09/25.

24. Georgalas I, Pavesio C, Ezra E. Bilateral cystoid macular edema in a patient with chronic myeloid leukaemia under treatment with imanitib mesylate: report of an unusual side effect. Graefes Arch Clin Exp Ophthalmol. 2007;245(10):1585-6. Epub 2007/06/15.

25. Ho WL, Wong H, Yau T. The ophthalmological complications of targeted agents in cancer therapy: what do we need to know as ophthalmologists? Acta Ophthalmol. 2013;91(7):604-9. Epub 2012/09/14.

26. Breccia M, Gentilini F, Cannella L, Latagliata R, Carmosino I, Frustaci A, et al. Ocular side effects in chronic myeloid leukemia patients treated with imatinib. Leuk Res. 2008;32(7):1022-5. Epub 2007/12/07.

27. Hu H, Hao L, Yan B, Li X, Zhu Y, Yao J, et al. Gefitinib inhibits retina angiogenesis by affecting VEGF signaling pathway. Biomed Pharmacother. 2018;102:115-9. Epub 2018/03/20.

28. electronic medicines compendium e. Product Information: ganciclovir sodium powder for concentrate for solution for infusion. United Kingdom2016 [updated 15.12.2020; cited 2021 05.03.2021]; Summaries of Product Characteristics]. Available from: https://[www.medicines.org.uk/emc/product/10242/smpc](http://www.medicines.org.uk/emc/product/10242/smpc).

29. Berdugo M, Delaunay K, Naud MC, Guegan J, Moulin A, Savoldelli M, et al. The antidiabetic drug glibenclamide exerts direct retinal neuroprotection. Transl Res. 2021;229:83-99. Epub 2020/10/21.

30. Iqbal Y, Palkar V, Al-Sudairy R, Al-Omari A, Abdullah MF, Al-Debasi T. Papilledema, presenting as reversible loss of vision, in a child with acute lymphoblastic leukemia. Pediatr Blood Cancer. 2005;45(1):72-3. Epub 2005/03/15.

31. Sharma P, Sharma R. Toxic optic neuropathy. Indian J Ophthalmol. 2011;59(2):137-41. Epub 2011/02/26.

32. Balachandran C, McCluskey PJ, Champion GD, Halmagyi GM. Methotrexate-induced optic neuropathy. Clin Exp Ophthalmol. 2002;30(6):440-1. Epub 2002/11/13.

33. Sbeity ZH, Baydoun L, Schmidt S, Loeffler KU. Visual field changes in methotrexate therapy. Case report and review of the literature. J Med Liban. 2006;54(3):164-7. Epub 2006/12/28.

34. Kawaji T, Inomata Y, Takano A, Sagara N, Inatani M, Fukushima M, et al. Pitavastatin: protection against neuronal retinal damage induced by ischemia-reperfusion injury in rats. Curr Eye Res. 2007;32(11):991-7. Epub 2007/11/21.

35. Monge KS, Galvez-Ruiz A, Alvarez-Carron A, Quijada C, Matheu A. Optic neuropathy secondary to dasatinib in the treatment of a chronic myeloid leukemia case. Saudi J Ophthalmol. 2015;29(3):227-31. Epub 2015/07/15.

36. Gordon B, Chang S, Kavanagh M, Berrocal M, Yannuzzi L, Robertson C, et al. The effects of lipid lowering on diabetic retinopathy. Am J Ophthalmol. 1991;112(4):385-91. Epub 1991/10/15.

37. Griffin JD, Garnick MB. Eye toxicity of cancer chemotherpay: a review of the literature. Cancer. 1981;48(7):1539-49.

38. Noureddin BN, Seoud M, Bashshur Z, Salem Z, Shamseddin A, Khalil A. Ocular toxicity in low-dose tamoxifen: a prospective study. Eye (Lond). 1999;13 ( Pt 6):729-33. Epub 2000/03/09.

39. Hwang N, Chung SW. Sulfasalazine attenuates tamoxifen-induced toxicity in human retinal pigment epithelial cells. BMB Rep. 2020;53(5):284-9. Epub 2020/04/23.

40. Kaewkhaw R, Rojanaporn D. Retinoblastoma: Etiology, Modeling, and Treatment. Cancers (Basel). 2020;12(8). Epub 2020/08/23.
